# The Importance of Veterinary Policy in Preventing the Emergence and Re-Emergence of Zoonotic Disease: Examining the Case of Human African Trypanosomiasis in Uganda

**DOI:** 10.3389/fpubh.2014.00218

**Published:** 2014-11-03

**Authors:** Anna L. Okello, Susan C. Welburn

**Affiliations:** ^1^Division of Pathway Medicine, Centre for Infectious Diseases, School of Biomedical Sciences, College of Medicine and Veterinary Medicine, The University of Edinburgh, Edinburgh, UK

**Keywords:** veterinary services, neglected zoonotic diseases, public health, human African trypanosomiasis, Uganda, veterinary policy

## Abstract

Rapid changes in human behavior, resource utilization, and other extrinsic environmental factors continue to threaten the current distribution of several endemic and historically neglected zoonoses in many developing regions worldwide. There are numerous examples of zoonotic diseases which have circulated within relatively localized geographical areas for some time, before emerging into new regions as a result of changing human, environmental, or behavioral dynamics. While the world’s focus is currently on the Ebola virus gaining momentum in western Africa, another pertinent example of this phenomenon is zoonotic human African trypanosomiasis (HAT), endemic to south and eastern Africa, and spread via infected cattle. In recent years, the ongoing northwards spread of this disease in the country has posed a serious public health threat to the human population of Uganda, increasing the pressure on both individual families and government services to control the disease. Moreover, the emergence of HAT into new areas of Uganda in recent years exemplifies the important role of veterinary policy in mitigating the severe human health and economic impacts of zoonotic disease. The systemic challenges surrounding the development and enforcement of veterinary policy described here are similar across sub-Saharan Africa, highlighting the necessity to consider and support zoonotic disease control in broader human and animal health systems strengthening and associated development programs on the continent.

## Introduction

Human African trypanosomiasis (HAT or sleeping sickness) is classified by the World Health Organization (WHO) as a neglected zoonotic disease (NZD). Endemic across sub-Saharan Africa, HAT is transmitted to human beings through bites from the *Glossina* species of tsetse fly. Although not a novel or recently emerging disease, zoonotic HAT has been described as the “avian flu of its time” (1), associated with devastating epidemics and emergence into previously naive areas, as recently observed in Uganda (2). HAT is therefore an interesting case study through which to examine the impact of veterinary policy in mitigating the emergence and re-emergence of zoonoses into new regions; a topical issue given the unfolding Ebola crisis in parts of western Africa and other “worrying developments (*that*) show it might be time to reassess the old ideas about the distribution of these (*neglected tropical*) diseases” (3). The first purpose of this paper is to provide a short review of the history of HAT in Uganda, in order to contextualize the subsequent exploration of the factors that impede or facilitate the refinement and enforcement of veterinary policies in the country. Finally, the discussion of available options and recommendations to prevent further emergence and re-emergence of HAT in the country can be applied to zoonotic diseases more generally across the continent.

There are two forms of HAT, their geographic boundaries roughly separated by the Rift Valley. The acute, zoonotic *Trypanosoma brucei rhodesiense* form is found in eastern and southern Africa, and the chronic, non-zoonotic *Trypanosoma brucei gambiense* form occurs throughout western Africa. Animal African trypanosomiasis (AAT or “nagana”) is the corresponding syndrome in livestock, caused by various species of trypanosome also transmitted by the *Glossina* tsetse fly. Tsetse and trypanosomiasis control and eradication programs in Africa have a long history, dating from colonial times when European powers were concerned with human epidemics and the loss of animal productivity associated with the disease (4). Control efforts were largely successful up until the 1960s; however, human cases have been rising steadily since independence, with 50–60 million people across the continent currently exposed to the bite of a tsetse fly (5). Despite “political will at the highest levels” to resurrect collective action for the control of human and AAT in Africa, many feel that there is still a long way to go before the disease regains the attention it deserves and reduces to the pre-1960s level (6, 7). The intersectoral approach required for the control of tsetse and trypanosomiasis “lies at the heart of African rural development”; shown to benefit the livelihoods of the rural poor both directly through improved health and increased nutritional outputs of livestock and indirectly through improved agricultural productivity and subsequent food security via provision of livestock outputs such as draught power and manure (5, 8).

Sharing symptoms with malaria and HIV/AIDs, HAT is often misdiagnosed or underreported by health authorities, with estimations that of the 300,000 new cases of HAT every year, only 30,000–40,000 are recorded because of issues with accessibility and quality of health services, particularly in rural areas (5). With vague first stage symptoms including recurring fever, joint pain, and nausea, the diagnosis of second stage disease is even more difficult in the absence of trained professionals, given the requirement for the trypanosome parasite to be detected in a sample of cerebrospinal fluid. Once diagnosis is made, the treatment (particularly for *T. br. rhodesiense*) is severe, with death, resulting in around 10% of cases. Without treatment, however, patients infected with both the acute and chronic forms of HAT will likely die. The current gaps in funding and technology for HAT diagnosis and treatment are a telling indicator of its “neglected” status; in the case of *T. br. rhodesiense*, no new drugs have been developed for over 60 years.

## “A Sense of Urgency”: Recent Emergence of Acute Human African Trypanosomiasis in Central and Northern Uganda

Uganda is presently the only country to harbor foci of both the acute and chronic forms of HAT, with a focus of *T. br. gambiense* in the West Nile region to the northwest, and *T. br. rhodesiense* endemic across the southeast region of Busoga. The initial emergence of *T. br. rhodesiense* HAT in Uganda was thought to have occurred as a result of European invasion along the Congo River to Lake Victoria, with *T. br. gambiense* entering the country as a result of human migration from central to western Africa (9). Although tsetse flies have been in Uganda for “thousands of years,” the public health impact and subsequent academic interest in the disease started during the early twentieth century, when it was estimated a third of Uganda’s population died of acute HAT in burgeoning epidemics (9).

Progression in molecular technologies toward the end of the twentieth century established the domestic cattle population as the most significant animal reservoir in Uganda, essential for the maintenance of *T. br. rhodesiense* within human populations (10–12). Major HAT epidemics in Uganda’s history have been associated with large cattle losses; over a million Ugandan cattle died in the 1890s, Rinderpest outbreaks across the country, resulting in large tracts of overgrown grazing land conducive to escalating tsetse infestations. It is thought that the tsetse flies “ran short” of cattle to feed on during this time, reverting to human blood meals, which perpetuated the spread of disease during this period (9). More recently, northwards spread of HAT has been associated with conflict and lord’s resistance army (LRA) insurgence from the 1980s; people fled their homes, taking their livestock with them, which led to overgrowth of tsetse territory; “By 1990, there were only roughly one thousand cattle left in Soroti district as most of the cattle had died because of diseases, been rustled or eaten by the armed groups in the different conflicts … growth of forests as a result of the war attracted the tsetse flies … sleeping sickness was rampant” (9).

Recent net migration of human beings and livestock back into previously overgrown areas has been attributed to this northwards spread of disease during the latter part of the 1990s (11, 13, 14). Mass rural development programs, funded by both the Ugandan government and foreign donors, were implemented upon the return of civic stability to several districts in central and northern Uganda during the 1990s. A major re-stocking exercise commenced, promoting the return of cattle and other livestock back into the area to assist the resumption of agro-pastoral activities. By 2005, however, there was a public health crisis in Uganda; molecular technologies indicated acute *T. br. rhodesiense* HAT had spread into eight new districts in as many years, with only 150 km separating the acute and chronic foci of human disease (2). The spread of disease is thought to be largely attributed to cattle movement from infected *T. br. rhodesiense* areas in the southeast of the country to previously free areas further north. It is feared that overlap of the two disease foci will spark a public health nightmare; given the parasites are morphologically similar on blood and cerebrospinal fluid smears, the only way to determine the appropriate therapeutic treatment is to know which geographical area the human patient comes from. Furthermore, the clinical effects of mixed infection, and the optimal treatment protocol, are currently unknown.

## Methodology

Combination qualitative research consisting of semi-structured interviews (SSIs), focus group discussions (FGDs), and observation techniques was recently undertaken in Uganda, in order to understand current veterinary policies and their level of enforcement, particularly regarding pre-movement treatment of livestock in HAT endemic areas. Key informant interviews (*n* = 13) were conducted with ministerial representatives and policy officers in the Ministries of Health and Agriculture at both the central and district government levels. In addition, field visits were undertaken in Soroti and Serere Districts in north-central Uganda, where HAT cases had recently emerged. Observations at local livestock markets complemented FGDs (*n* = 16) with farmers and local animal health officers. All key informant interviews were conducted in English, with the FGDs conducted in a mixture of Ateso and English, facilitated by a translator. The resulting transcripts were entered into Microsoft Word 2010 and manually coded according to themes, ideas, and opinions in order to develop the ensuing narratives presented in this manuscript.

## Results

A major objective of this research was to identify and analyze the current livestock movement and disease control policies that exist in Uganda, and subsequently their level of enforcement through direct observations and data collection at the district level. The major policy concerning zoonotic disease control within animal reservoirs in Uganda is the Animal Disease Act (1918), which describes the requirements for addressing outbreaks of notifiable zoonoses such as rabies, anthrax, and trypanosomiasis (15). A separate Veterinary Public Health Act exists to promote meat inspection and milk hygiene for the control of food-borne zoonoses such as brucellosis and bovine tuberculosis. Both acts are in need of updating, however, at present “*these are the policies that are followed as no new ones have been written post-independence*” (Informant interview, government sector).

Specifically concerning livestock movement, the official policy within the 1918 Animal Disease Act states animals cannot be moved into new areas “without clearance from veterinary officers” (15). These livestock movement restrictions were further revised after the research linking cattle movement and spread of human disease was published, resulting in new policy requiring pre-movement trypanocidal treatment of all cattle in endemic regions (16). The current procedure that should therefore be followed when cattle move from HAT endemic areas in the country’s southeast is contained within Section 18 of Uganda’s Animal Disease Act entitled *Rules for Infected Areas*, with particular reference to the following items:

Item 1: no stock or carcass shall be moved in or from any such area without the written permission of the commissioner of livestock and entomology or the veterinary officer or inspecting officer in charge of the area.
Item 6: no person shall leave any such area without having complied with such precautions for preventing the spread of disease as may be required by the veterinary officer or inspecting officer in charge of the area.

A second objective of the ongoing HAT research in Uganda is to establish the extent to which this existing pre-movement treatment policy is currently enforced at district livestock markets in Uganda, particularly given its importance in decreasing disease into new areas and across international borders. When asked whether they have witnessed government veterinarians treating animals at the market places, the majority of farmers indicated this was done “*from time to time*,” however, some were suspicious of veterinarians under-dosing “*as the dose is very small*” (Focus Group Participants, Serere and Soroti Districts, Uganda). Most respondents signified they had to pay for treatment (between 1000 and 5000 USh) and received certificates; “*there is a doctor there, after you buy the cow, they tell us that they are treating for a certain disease, and it is a must; we cannot go without them treating our cow, it is under order*” (Focus Group Participant, Serere District, Uganda). When probed, few individuals knew what treatment their animals were receiving, justifying their lack of knowledge as “*the government vets are trying to reduce the diseases; they themselves know what they are treating for*” (Focus Group Participant, Soroti District, Uganda). Although this may be true to some extent, the current lack of community sensitization to HAT severity and its transmission is an issue; if farmers are not aware of the linkages between their cattle and *T. br. rhodesiense*, they will not see the importance for continuous post-treatment spraying, and could be further at risk of contracting the disease from their cattle in endemic areas.

A further interesting observation was made regarding the strict enforcement of livestock movement restrictions during an outbreak of Foot and Mouth Disease (FMD) in the area at the time of research (Anna L. Okello, field observations). In this way, it seems that despite the human and financial resource issues in the wider veterinary systems in Uganda, enforcement of veterinary policy is indeed possible where political motivation exists. Despite the reasons for quarantine being largely understood and respected by farmers, it was evidently placing a strain on livelihoods in the area; focus group accounts of meager cash flows as a result of livestock market closures were echoed by a private veterinary service provider in the district; “*(the quarantine) has been devastating for families and this time it has collided with going back to school – parents have no money for school fees or books, and this has been reflected in the low school attendance*” (Key informant interview, Serere District, Uganda). Some farmers explained how the lack of available animals for dowry effectively ceased marriages in the district; “*nobody is getting married – we have been told to hold off the marriage until the quarantine lifts*” (Focus Group Participant, Soroti District, Uganda).

## Discussion

A major driver of the recent northwards movement of *T. br. rhodesiense* is the ongoing northern migration of cattle from endemic regions in the southeast, particularly since the opening up of the central and northern regions after decades of conflict (11, 13, 14). While this case study focuses on HAT, it must be remembered that this is not the only potentially fatal zoonotic disease to emerge or re-emerge as a result of infected animals entering regions where it has been previously controlled; brucellosis, rabies, porcine cysticercosis, and bovine tuberculosis are all spread through poor enforcement of veterinary policies regarding abattoir inspection, inadequate attention to disease control, and unregulated regional trade. It is for this reason that understanding the current levels of enforcement of veterinary policy, and the bottlenecks regarding this, is important.

In Uganda, a commonly cited reason for the delayed revision of the outdated veterinary policy concerns the lack of evidence – particularly prevalence data – for disease prioritization; “*you need to provide information on what the problem is, the nature of transmission, its economic and public health importance – then you can bring the stakeholders on board for their views*” (Key informant interview). For the majority of neglected tropical diseases, however, there is often difficulty in securing funds for prevalence studies in the first place; “*as much as you don’t want a political crisis, we need the data for justification of spending … We are all fighting for meagre resources*” (Key informant interview).

Second, limited financial and political focus on veterinary services across much of sub-Saharan Africa has resulted in severe systemic sectoral weaknesses. For this reason, while the policy may exist, the enforcement does not, as was observed in Uganda; “*we have a very broad Animal Disease Act, which is the major policy document that directs disease control in the country, and it has provisions for most of those things. The current problem is with the implementation*” (Informant interview, government sector). Poor adherence to national policy advising pre-movement treatment of cattle with trypanocidal drugs prior to their removal from *rhodesiense*-endemic areas in the southeast of Uganda has therefore likely played a role in the northwards spread of disease in recent years. Some have also indicated the insufficient technical input into the re-stocking movement, and its highly politicized nature – “*the re-stocking deal was done in the office of the Prime Minister*” (Informant interview, government sector) – may have inadvertently exacerbated the issue even further. This lack of opportunity for technocrats to input into the policy process has been well documented in sub-Saharan Africa (17).

At the ground level, there was further evidence that enforcement of current veterinary policy differed largely according to geographical location, likely reflecting individual commitment and available veterinary resources in that particular district. For example, while Kaberamaido and Serere Districts appeared to enforce stricter protocols surrounding pre-treatment movement of cattle, others did not; “*treatment occurred long ago but not now, we’ve not seen anything the past few years*” (Focus group participant, Soroti District, Uganda). The requirement for livestock movement policy enforcement is even more urgent given the propensity for farmers to prioritize cattle sales when they know international buyers and donor re-stocking programs are in operation; “*when the traders arrive from Sudan, (we) get good prices. They’re taken by truck to Sudan, far away. And also the NGOs and government (for re-stocking); they give good prices, the community knows when to sell through word of mouth*” (Focus Group, Serere District, Uganda). In general, the documented field observations and farmer experiences support recently published concerns that “(Ugandan veterinary policy) implementation continues to be very patchy, with some traders and even some NGOs wholly bypassing controls” (18).

The other major observations concern the limited community level advocacy regarding the dangers of *T. br. rhodesiense* infected cattle, and the need for enabling policies that promote the required preventative animal husbandry at the farm level. In human beings, HAT’s similar clinical presentation to higher profile diseases such as malaria and HIV contributes to poor public awareness; “*people are dying from sleeping sickness, but they were thinking it was HIV/AIDS, because that thing can also make you become very thin, so some people were just left to die like that*” (Informant interview Serere District, Uganda 2011). Similarly for livestock, while the various animal-specific *Trypanosome* species result in a clinically ill animal, *T. br rhodesiense* does not have any clinical impact, thus, resulting in even less desire for farmers to spend money treating what they essentially view as a “healthy” animal. The current evidence indicates that farmers prioritize tick control, given the visibility of these parasites on stock and the known impact of tick-borne diseases such as East Coast Fever (ECF). While dual-preparations that act on both ticks and tsetse flies are registered for livestock in Uganda, they are more expensive and less readily available, so smallholder farmers are less inclined to use them. Most farmers interviewed purchase the cheaper tick-only preparations that have no impact on *Trypanosome* transmission, consistent with previous findings by Bardosh et al. (19). Improving the supply and economic justification from both the human and animal health perspective for why farmers should use tsetse-tick preparations would be an important way forwards for the Ugandan veterinary sector, and one that is recommended by the authors.

Finally, it is interesting to note that the Animal Disease Act can be enforced for trade diseases such as FMD, despite in this case the socioeconomic impact of quarantine appearing arguably greater than the disease itself, given the limited access of smallholder farmers to formal livestock markets. Considering “pro-eradication” policies for the control of trade diseases such as FMD are able to remain strong in this way, it is vital that the same attention is turned to zoonoses control in endemic countries, to prevent further emergence into naive areas or across international borders.

Through documenting the recent observations of HAT control in Uganda, we have highlighted the role of the veterinary sector in zoonoses prevention, presenting a case for greater economic and institutional focus on the African veterinary sector as a whole. However, long distances, poor infrastructure, and limited cash flow/disposable income all impact the provision and utilization of quality human and animal health services across much of sub-Saharan Africa. This ultimately affects disease reporting, impacting the level of attention placed on a disease issue by policy makers (20). Furthermore, even where policy actors do accept the need for zoonoses control, the issues of policy development and enforcement are compounded where multiple sectors are involved, further impeding the timely adoption and implementation of innovative policy processes for zoonoses control (17). It is for this reason that veterinary sector improvements need to consider the various external economic and developmental bottlenecks to policy development, enforcement, and control that could impact disease control more generally on the continent.

## Conclusion

There are several ongoing policy issues regarding livestock movement and disease control in Uganda, stemming from a variety of social, economic, and political factors. Up to date, enforceable veterinary policy has the potential to significantly contribute to the economic development of a country via joint impacts on human and animal disease control, improved production outputs that impact regional markets, and access of the human population to improved nutrition. Despite this, the importance of veterinary policy is often underestimated by the health and development sectors alike, particularly regarding its role in the prevention of emerging and re-emerging zoonotic disease, as described by this case study.

Weak enforcement of a veterinary policy directive that simultaneously controls a fatal human disease and improves livestock productivity questions the type of evidence, advocacy, and capacity required to ensure zoonoses control efforts in animal reservoirs are realized. We need to overcome this disabling triad of capacity, advocacy, and evidence in order to move beyond our relatively limited understanding of zoonoses transmission – and its implications in changing agricultural contexts – toward a fuller, holistic, and more responsive veterinary system of analysis, intervention, and control. Awareness of the potentially harmful human diseases carried by domestic animals and wildlife is vitally important, not only just for the communities that face daily exposure to them but also for the policy actors and key decision-makers that play a role in preventing the emergence and spread of zoonoses at the crossroads of human health, agriculture, and development.

## Conflict of Interest Statement

The authors declare that the research was conducted in the absence of any commercial or financial relationships that could be construed as a potential conflict of interest.

## References

[1] Okello AL Bardosh K Smith J Welburn SC. One health: past successes and future challenges in three African contexts. PLoS Negl Trop Dis (2014) 8(5):e2884. 10.1371/journal.pntd.0002884 24851901 PMC4031173

[2] Picozzi K Fèvre EM Odiit M Carrington M Eisler MC Maudlin I Sleeping sickness in Uganda: a thin line between two fatal diseases. Br Med J (2005) 331:1238–41. 10.1136/bmj.331.7527.1238 16308383 PMC1289320

[3] The Lancet Infectious Diseases. Neglected tropical diseases: no longer someone else’s problem. Lancet Infect Dis (2014) 14(10):899. 10.1016/S1473-3099(14)70928-4 25253395

[4] Schofield C Kabayo JP. Trypanosomiasis vector control in Africa and Latin America. Parasit Vectors (2008) 1:24. 10.1186/1756-3305-1-24 18673535 PMC2526077

[5] Cattand P Simarro P Jannin J Ly C Shaw A Mattioli R. Linking Sustainable Human and Animal African Trypanosomiasis Control With Rural Development Strategies. PAAT Technical and Scientific Series, No. 10. ISBN 978-92-5-106670-6. Rome: Food and Agriculture Organization (2010). Available from: http://www.fao.org/docrep/013/i1790e/i1790e00.pdf

[6] Molyneux D Ndung’u J Maudlin I. Controlling sleeping sickness – when will they ever learn? PLoS Negl Trop Dis (2010) 4(5):e609. 10.1371/journal.pntd.0000609 20520799 PMC2876118

[7] Simarro PP Jannin J Cattand P. Eliminating human African trypanosomiasis: where do we stand and what comes next? PLoS Med (2008) 5(2):e55. 10.1371/journal.pmed.0050055 18303943 PMC2253612

[8] Kristjanson PM Swallow BM Rowlands GJ Kruska RL de Leeuw PN. Measuring the costs of African animal trypanosomiasis, the potential benefits of control and returns to research. Agric Syst (1999) 59:79–98. 10.1016/S0308-521X(98)00086-9

[9] Waiswa C Kabasa JD. Historical mapping of events in the SOS districts. *Department for International Development Research Into Use Programme (2006–2012) Project Documents*. Kampala (2009).

[10] Hide G Tait A Maudlin I Welburn SC. The origins, dynamics and generation of *Trypanosoma brucei rhodesiense* epidemics in East Africa. Parasitol Today (1996) 12:50–5. 10.1016/0169-4758(96)80654-5 15275254

[11] Fèvre EM Coleman PG Odiit M Magona JW Welburn SC Woolhouse ME. The origins of a new *Trypanosoma brucei rhodesiense* sleeping sickness outbreak in eastern Uganda. Lancet (2001) 358:625–8. 10.1016/S0140-6736(01)05778-6 11530149

[12] Welburn SC Fèvre EM Coleman PG Maudlin I. Epidemiology of human African trypanosomiases. In: Maudlin I Holmes P Miles M, editors. The Trypanosomiases. Wallingford: CABI Publishing (2004). p. 219–32

[13] Welburn SC Coleman PG Maudlin I Fèvre EM Odiit M Eisler MC. Crisis, what crisis? Control of Rhodesian sleeping sickness. Trends Parasitol (2006) 22:123–8. 10.1016/j.pt.2006.01.011 16458071

[14] Selby R Bardosh K Picozzi K Waiswa C Welburn SC. Cattle movements and trypanosomes: restocking efforts and the spread of *Trypanosoma brucei rhodesiense* sleeping sickness in post-conflict Uganda. Parasit Vectors (2013) 6:281. 10.1186/1756-3305-6-281 24289452 PMC3851531

[15] Government of Uganda Animal Diseases Act. (2014). Available from: http://www.ulii.org/ug/legislation/consolidated-act/38

[16] Wendo C. Uganda revises cattle treatment to protect humans from sleeping sickness. Lancet (2002) 359:239. 10.1016/S0140-6736(02)07489-5 11812574

[17] Okello AL Welburn SC Smith J. Crossing institutional boundaries: mapping the policy process for improved control of endemic and neglected zoonoses in sub-Saharan Africa. Health Policy Plan (2014). 10.1093/heapol/czu059 25000963

[18] Morton J. The innovation trajectory of sleeping sickness control in Uganda: research knowledge in its context. Research into Use Discussion Paper 08 (2010). Available from: http://www.researchintouse.com/resources/riu10discuss08ssickcntrl-ug.pdf

[19] Bardosh K Waiswa C Welburn SC. Conflict of interest: use of pyrethroids and amidines against tsetse and ticks in zoonotic sleeping sickness endemic areas of Uganda. Parasit Vectors (2013) 6:204. 10.1186/1756-3305-6-204 23841963 PMC3711891

[20] Maudlin I Eisler MC Welburn SC. Neglected and endemic zoonoses. Philos Trans R Soc Lond B Biol Sci (2009) 364:2777–87. 10.1098/rstb.2009.0067 19687045 PMC2865085

